# Lessons From a Rapid Project Management Exercise in the Time of Pandemic: Methodology for a Global COVID-19 VIRUS Registry Database

**DOI:** 10.2196/27921

**Published:** 2022-03-15

**Authors:** Janice R Turek, Vikas Bansal, Aysun Tekin, Shuchita Singh, Neha Deo, Mayank Sharma, Marija Bogojevic, Shahraz Qamar, Romil Singh, Vishakha Kumar, Rahul Kashyap

**Affiliations:** 1 Department of Management Engineering and Consulting Mayo Clinic Rochester, MN United States; 2 Division of Pulmonary and Critical Care Medicine Mayo Clinic Rochester, MN United States; 3 Department of Anesthesiology and Perioperative Medicine Mayo Clinic Rochester, MN United States; 4 The Society of Critical Care Medicine Mount Prospect, IL United States

**Keywords:** COVID-19, critical care, global, program management, registry

## Abstract

**Background:**

The rapid emergence of the COVID-19 pandemic globally collapsed health care organizations worldwide. Incomplete knowledge of best practices, progression of disease, and its impact could result in fallible care. Data on symptoms and advancement of the SARS-CoV-2 virus leading to critical care admission have not been captured or communicated well between international organizations experiencing the same impact from the virus. This led to the expedited need for establishing international communication and data collection on the critical care patients admitted with COVID-19.

**Objective:**

Developing a global registry to collect patient data in the critical care setting was imperative with the goal of analyzing and ameliorating outcomes.

**Methods:**

A prospective, observational global registry database was put together to record extensive deidentified clinical information for patients hospitalized with COVID-19.

**Results:**

Project management was crucial for prompt implementation of the registry for synchronization, improving efficiency, increasing innovation, and fostering global collaboration for valuable data collection. The Society of Critical Care Medicine Discovery VIRUS (Viral Infection and Respiratory Illness Universal Study): COVID-19 Registry would compile data for crucial longitudinal outcomes for disease, treatment, and research. The agile project management approach expedited establishing the registry in 15 days and submission of institutional review board agreement for 250 participating sites. There has been enrollment of sites every month with a total of 306 sites from 28 countries and 64,114 patients enrolled (as of June 7, 2021).

**Conclusions:**

This protocol addresses project management lessons in a time of crises which can be a precept for rapid project management for a large-scale health care data registry. We aim to discuss the approach and methodology for establishing the registry, the challenges faced, and the factors contributing to successful outcomes.

**Trial Registration:**

ClinicalTrials.gov NCT04323787; https://clinicaltrials.gov/ct2/show/NCT04323787

## Introduction

The COVID-19 pandemic has introduced unprecedented challenges to health care systems worldwide [1]. Because of the effects of COVID-19 on the respiratory system [2], geographic areas affected by the pandemic have experienced large surges in critically ill patients who require intensive care and multiple organ system support [3]. The recognition for the necessity of conception of a COVID-19 global critical care database rapidly developed with the growing crisis hospitals experienced from the spread of the disease [4,5]. This would allow for near real-time data collection, analysis, and display. The design of the registry would be consistent with data analytic requirements [4,5]. Rapid formation of partnerships, gaining needed resources, identifying and enrolling participants, and developing the tool to collect data are crucial and time sensitive due to the rapid progression of the virus.

Implementation of a global registry during a pandemic requires extensive project management, which includes planning, initiation, execution, monitoring, and eventually closing of the registry [6,7]. Early steps require (1) research approvals for human patients/participants and data use agreements; (2) development of electronic case report forms; (3) defining common data standards and terminology; (4) development of standard operating procedure (SOP) and training for data entry; (4) coordination with other studies; (5) data quality control, automation, and validation; and finally (6) planning for diverse methods of knowledge dissemination through the registry dashboard and publications.

Project management, in a methodical but agile approach, is fundamental to release a data collection tool that would benefit health care organizations worldwide. The shared registry would enable organizations to review and analyze data in accelerated time frames on a continuous ongoing spectrum. Rapid project management and successfully implementing an initiative of this magnitude and complexity require strong leadership and the right partnerships [8,9]. An iterative, team-based approach without any lag time between different phases to get tasks done would be ideal in contrast to the waterfall methodology.

The Viral Infection and Respiratory Illness Universal Study (VIRUS): COVID-19 Registry [4,5] is a first of its kind, enrolling patients from 25 countries and more than 275 participating sites aimed at sharing real-time information on hospital care and intensive care unit admissions, which could expand the scope of SARS-CoV-2 research. The objective of this protocol was to report the project management activities, timelines, and various steps for implementation of the COVID-19 global registry. We aim to discuss the outcomes, factors which led to successful implementation, and challenges faced which could act as a guide for future establishment of large-scale registries in times of acute need.

## Methods

### Ethics Approval

The VIRUS Registry was approved by the Mayo Clinic Institutional Review Board (IRB; approval no. 20-002610). The Mayo Clinic IRB waived the need to obtain consent for the collection, analysis, and publication of the anonymized data for this non-interventional study and determined to be exempt from the requirement for IRB approval (45 CFR 46.104d, category 4).

### Overview of the Society of Critical Care Medicine Discovery VIRUS: COVID-19 Registry

The detailed methodology and aims of the registry have been published elsewhere [4,5]. In brief, the VIRUS Registry [4,5] is a prospective, observational global registry database. In this registry, except 25 sites in the United States with data automation, all other sites collect data manually on a voluntary basis. Case report forms were constructed using Research Electronic Data Capture (REDCap) [10], to record deidentified clinical information as well as daily physiologic, laboratory, and treatment information for patients hospitalized with COVID-19.

### Project Management Review

#### Team Organization Structure

**Figure 1:**
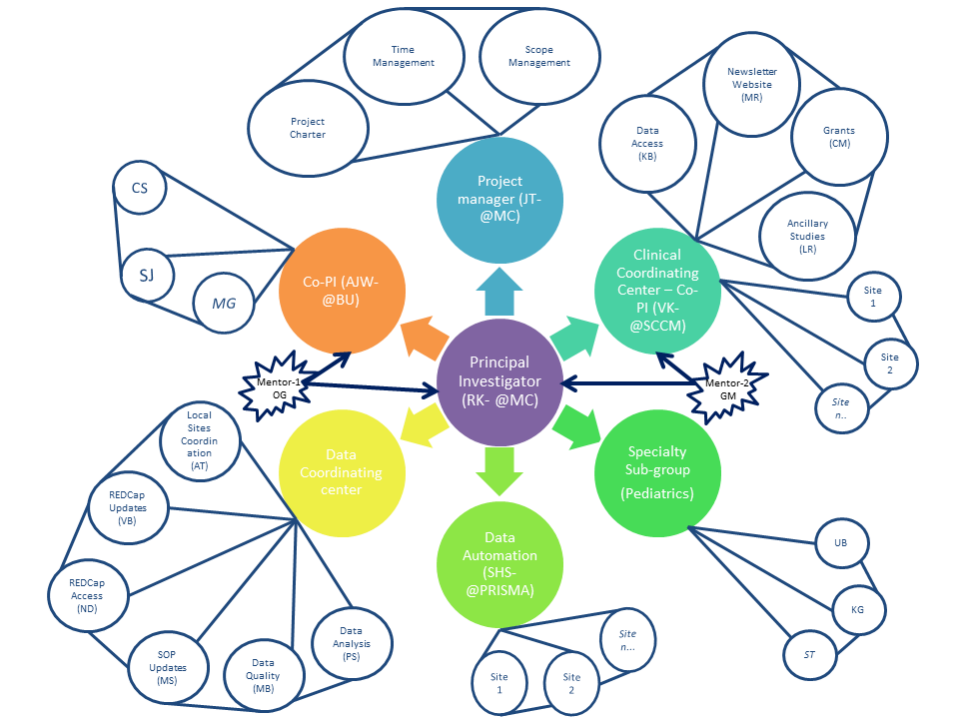
VIRUS Global Pandemic Registry Team Organizations. AJW: Allan J Walkey. AT: Aysun Tekin. BU: Boston University. CM: Colleen McNamara. CS: Christopher R Sheldrick. GM: Gregory Martin. JT: Janice Turek. KB: Karen Boman. KG: Katja Gist. LR: Lynn Retford. MB: Marija Bogojevic. MC: Mayo Clinic. MG: Michael Garcia. MR: Mary Reidy. MS: Mayank Sharma. ND: Neha Deo. OG: Ognjen Gajic. PS: Phillip Schulte. RK: Rahul Kashyap. SHS: Smith Heavner. SJ: Shelsey Johnson. ST: Sandeep Tripathi. UB: Utpal Bhalala. VB: Vikas Bansal. VK: Vishakha Kumar.

The team organization works based on the “hub and spoke” model, where the centralization around the principle investigator (primary contact) with primary team leads promotes stability within the organization. Members are often cross-trained and learn how to work with others outside of their immediate department. This team organization style was most suited for our needs and effective communications with each node (secondary contacts). Everyone is granted equal access to information, which promotes resource sharing and the “hub and spoke” distributed style was utilized at team member level (tertiary contacts; Figure 1). The model was generated by the tool provided from [11] (also see [12]). A blank template is provided in Multimedia Appendix 1.

#### Project Management

The first and imperative step in project management is quickly establishing a manageable scope [7]. The scope (Table 1) determines the parameters of the data collection, enabling the project team to better focus efforts on setting up the registry.

The project governance is identified by the project manager and project proponents. A rapid stakeholder analysis is crucial for alliance with organizations with the same interest and familiarity with international collaboration and identification of executive leadership [12].

**Table 1:** Scope description of the VIRUS^a^ registry.

Scope	Description
In scope description and vision	Scope: Hospitalized patients with COVID-19 who have positivity in PCR (or other SARS-CoV-2) test or test result pending or only observational clinical data. Vision: Real-time COVID-19 registry of current intensive care unit and hospital care patterns to allow evaluation of safety and observational effectiveness of COVID-19 practices.
Out of scope description	Non-COVID-19–related admissions, COVID outpatients, any intervention, any biospecimen collection.

^a^VIRUS: Viral Infection and Respiratory Illness Universal Study.

#### Project Plan

Creating a project plan led by the project manager assures that predecessor and dependent tasks are accounted for [13]. The plan lists the key and ongoing tasks and reviewing these on a regular basis assures tasks are not being missed and resources are not over allocated. The plan accounts for using the agile project framework utilizing the sprint methodology, which is necessary to quickly create a plan for global enrollment and education.

Developing a project timeline outlining milestones ensures a quick progress review, and helps with identifying roadblocks [14]. The timeline sums up the progression of the project.

#### Project Charter

Creating a project charter is necessary but not a priority at the start of the project. The charter will serve as a valuable tool for recording methodology, history of the project and project goals, and sustainment activities. It aids in creating and supporting a budget and obtaining financial assistance. The charter for this project was developed approximately 2 months after the initiation. Project information, data, and timelines had all been recorded and saved to allow for a charter to be efficiently created and remain an agile document with additions and modifications as the project progresses.

#### Steps Included in Registry Implementation for Obtaining Data Inputs

##### Defining Data Elements and Weekly REDCap Update for Patient Data Enrollment

**Figure 2:**
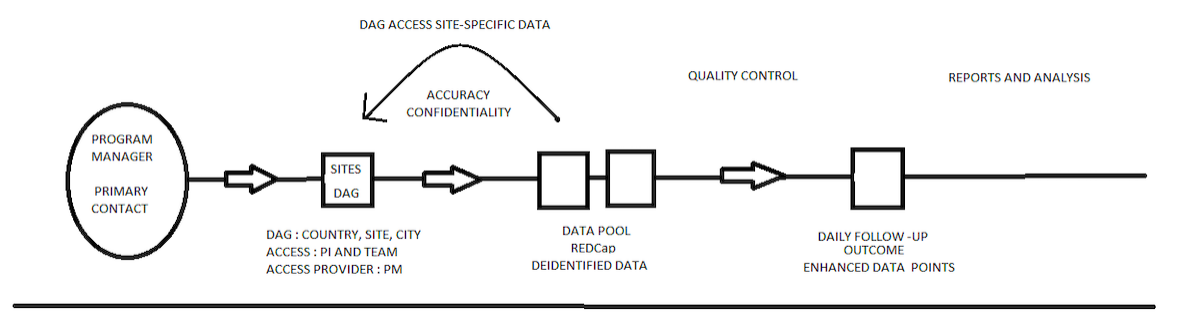
Workflow of the VIRUS: COVID-19 International registry. DAG: data access group, PI: program investigator, PM: program manager.

Focusing on data standards by using all existing standard data elements and definitions whenever possible is crucial for the development of interoperability and globalization of a registry aimed at consolidating data during a pandemic (Figure 2). This will be increasingly important as the use of electronic medical records is becoming widely available around the globe. It is also important to note that adopting standard data variables not only improves the efficiency in establishing registries but also promotes effective sharing, combining, or linking of data sets from different sources and institutions globally.

To achieve this, our registry adopted applicable data elements and definitions in accordance with (1) Critical Care Data Dictionary developed by the Data Definitions and Outcomes workgroup within the Society of Critical Care Medicine’s (SCCM) Discovery, the Critical Care Research Network; (2) available published data on COVID-19 standards; and (3) case report forms, data elements, and definitions from the World Health Organization (WHO) International Severe Acute Respiratory and Emerging Infection Consortium (ISARIC) COVID-19 core case report form.

##### Data Collection and Research Tools Management

Collecting and sharing data in a secure manner with numerous collaborators across academic departments or even institutions remain a formidable challenge. To address this challenge, the discovery and diagnosis phase of the project were combined and REDCap [10] was recognized as an optimal data collection tool for a large registry. REDCap is an electronic data capture system that allows electronic data securely while expediting the research process and ensuring data reusability. In addition, REDCap [10] allows real-time data validation, integrity checks, and other mechanisms to ensure data quality (eg, double-data entry options) and provides central data storage and backups.

##### REDCap Access Management

**Figure 3:**
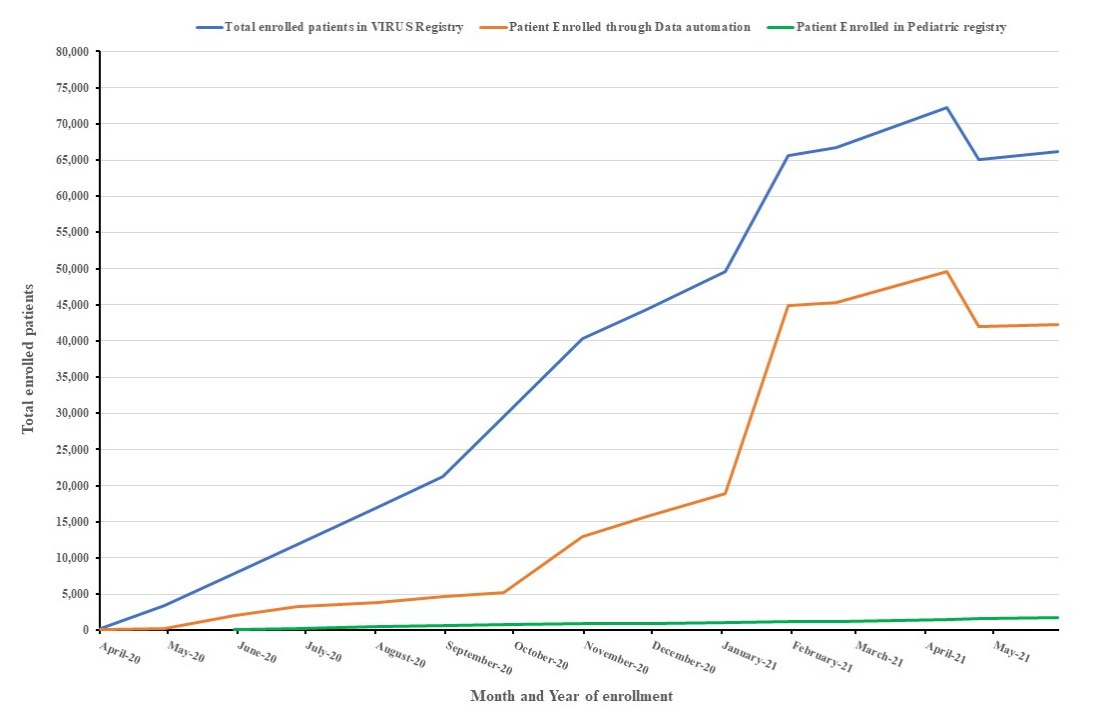
The one-year (April 2020-April 2021) trend of patient enrollment in VIRUS Registry.

REDCap access is necessary in order for each site to enter data for their patients. First, each hospital system needs to be inputted into REDCap as a data access group (DAG). A DAG only stores data for patients of that site. Each DAG is noted in the following format: country, site name, and city. Each site being labeled this way allows for easy organization and tracking of user accounts. To gain access to the respective DAG, either the principal investigator or members of the team request access to the data access manager. After requesting this, the data access manager requests an account creation for each respective user to REDCap administrators and then assigns them to the correct DAG. This ensures that the users in each team can only access patient data specific to their DAG, and not the rest of the patient database. This ensures accuracy and confidentiality of patient information (Figure 3).

##### Data Entry

Before entering data into the registry, it is important to define inclusion and exclusion criteria for the patients. Patients admitted to a participating hospital with COVID-19 were evaluated by the local institutional research teams according to the inclusion and exclusion criteria (Table 1). The process does not differ between floor or intensive care unit admissions. When included, each patient is assigned a study number and the real-time deidentified daily data collection begins thereafter. The data that are collected for each patient can be classified as baseline characteristics, daily follow-up, outcome, and enhanced data points. Detailed information regarding collected data points is outlined in Table 2. Patients were followed during the entire hospitalization period, regardless of any transfers between different divisions in the same institution. If a patient is transferred to another hospital, reenrollment does not occur, even if the new institution is participating in the registry. The outcome data are collected at the time of discharge from the hospital. On the 28th day after hospital admission, follow-up information regarding COVID-19 readmissions and 28-day mortality is recorded.

**Table 2:** Specifics of collected clinical data.

Data groups	Details
Baseline characteristics	Demographic characteristics Symptoms and history Comorbidities and premedications COVID-19 manifestations Microbiological test details
Daily follow-up	Imaging results Respiratory support details Medications and other interventions Employment of best care practices
Outcome	Requirement of oxygenation methods Complications during the follow-up period Hospital and intensive care unit length of stay In-hospital mortality 28-day mortality
Enhanced data point	Daily vital signs Comprehensive physical examination findings Detailed laboratory results Sequential Organ Failure Assessment (SOFA) and Pediatric Logistic Organ Dysfunction (PELOD) scores
Full data	Acute Physiological Assessment and Chronic Health Evaluation (APACHE) II score Electrocardiogram and echocardiography findings and deidentified image upload
Data regarding co-venting patients	Demographics of both patients Detailed ventilator setting information Outcome of both patients
Pediatric-specific data	Functional Status Score (FSS) and Pediatric Risk of Mortality (PRISM) scores

##### Personnel Training for Data Collection and Development of Standard Operating Procedure

Performing semistructured trainings with clinical research coordinators from participating study sites globally is very important to ensure good quality assurance. The goal of these meetings would be to provide a general overview of the registry database, while concurrently identifying specific factors which could compromise the integrity of data collection. In addition to structured training, a global registry requires a good SOP.

SOP is a set of detailed instructions that define and standardize research procedures in clinical registries. SOPs describe each data variable, standard definition and step of the research process, and the actions to be taken for data collection. It provides autonomy and improves the quality of the data collected, thereby improving the science of the study. SOP can be utilized as a reference and guideline as to how research will be conducted for new study sites, and to ensure the whole process is well described, comprehensive, predictable, less prone to error, and serves as an initial training source. Several iterations may be required before the needs and concerns of all are met. SOPs are a “living” instrument. They can only work if they reflect the actual process on the ground rather than what should ideally be happening. The document should be regularly updated to reflect changes or improvements in processes over time. Primary sites should retain and store older versions of an SOP to track what were done and what data were collected at what time and place.

To ensure a standardized and consistent data collection procedure, we developed a live SOP on Google Drive specifically related to the task of the primary data collectors. This SOP provides a description of all data elements collected as well as the sources used to obtain the data.

It is also crucial to have an oversight on data entry by experienced investigators. After the training process, the data entry activities of clinical research coordinators are closely monitored by the core team to assess whether data collection was conducted according to the study protocol.

##### Data Automation Management

Collaboration with health care centers to optimize electronic health record (EHR) data collection is a key in making data collection more efficient. During a pandemic, the primary concern of health care workers is to care for the patients, and therefore great effort needs to be made to make the process as smooth and efficient as possible. To manage data automation, we formed a working group called “Practical EHR Export Pathways” (PEEP). This group developed methods to automate the uploading of data from EHRs to reduce workloads from sites that were struggling with the heavy clinical burdens that had resulted because of the pandemic.

Recognizing data automation as an “intervention,” PEEP workgroup participating sites began an iterative process, with multiple sites producing and rapidly distributing resources (eg, MS Excel spreadsheet templates, SAS code, EHR–EPIC Workbench), incorporating feedback to describe successful tools, to optimize fit to practice settings and the ecological system, and to identify key constructs by consensus. PEEP workgroup–prioritized resources specific to the EPIC platform leverage standard search queries to obtain structured data from common EHR-generated relational databases and facilitate peer-to-peer coaching on the development and execution of end-user EPIC reporting functions. The participating sites reported implementation progress and validity through an online form and in biweekly team meetings.

##### Data Quality Management

The primary goal of the data quality checks is to ensure correct and consistent data entry [15]. The first step would be to analyze large volumes of data with attention to detail, accuracy, and data quality. This will help in creating acceptable data quality reports for any sort of registry. Troubleshooting to find root causes will also lead to quality improvement in the data collection tool. Preparation and consolidation of the data will be instrumental in periodic review and generation of data quality reports. The data collection tool (REDCap) also supports performing root cause analysis, investigating any data errors or anomalies, and assisting in implementing solutions to correct data problems. Meeting with study sites weekly and sharing inconsistencies and missing data for their sites will help in developing and publishing a set of quality metrics. It will facilitate capturing data trends on a weekly, monthly, and quarterly basis to ensure that data quality programs are working effectively.

##### Dissemination of Information Management

Partnering with a widely known medical organization or specialty society (for VIRUS registry, it was SCCM) with an imprint in the global health arena will prove to be a strategic measure enabling communication with worldwide organizations that enroll in the Registry. Here are a few highlights of communication management of the Registry:

- A communication plan needs to be put in place, identifying the standard content for each communication along with standard reporting.
- View of registry information needs to be granted to all participants so they could view information in real time.
- A public-facing webpage with registry information, contacts, and an intake form would be essential to manage the early barrage of inquiries.
- A weekly newsletter is the fastest and cheapest communication tool between the core team and participating sites.
- A public-facing data dashboard [16] would be key for simple descriptive data dissemination for participating investigators and other health care communities.
- Virtual meetings, weekly with all investigators and as needed with sites with various needs, would keep the communication lines open; details are described below.

##### Virtual Meetings

A pandemic usually results in a suspension of normal day-to-day activities. One of these activities is the group meeting among the researchers. Good communication between team members is key to having success in creating a registry. Therefore, it is essential to set up regular virtual meetings with clear agendas. To substitute for in-person meetings, since the beginning of the VIRUS: COVID-19 Registry project, 1-hour-long structured weekly meetings were put in place. Sharing information directly with the researchers from all study sites using Zoom as a tool for communication enables a sense of community and partnership with the global team [17]. The meeting agenda consists of updates regarding pediatric data, automation, adult data, and ancillary studies. Following these, a session takes place during which the participants get the opportunity to inquire about any unclear points. Content of the meeting sessions is summarized in Table 3.

**Table 3:** Project management significance: structure of the weekly meeting sessions.

Step/update	Specifics of the VIRUS^a^ Registry weekly follow-up	Significance to project management
Automation update	Data automation unit contributes with advancements in automated data collection. They also offer assistance and collaboration opportunities to participating institutions that are interested in automation.	Although there have been substantial developments in electronic health record–based automated data collection, there is still room for improvement. Not all sites may have the means for developing a feasible automation system all by themselves. Thus, the collaboration of sites under the supervision of a professional team helps individual sites to establish a system that would make reliable automated data collection possible.
Adult VIRUS registry data update	The project principal investigator brings the researchers up to date about the collected data. The study coordinating team provides comprehensive information about the updates in the standard operating procedure. They also indicate inconsistent and missing data points and share tips about improving the quality of collected data.	Acknowledgment of achievements, both for the whole project and for each site, increases the motivation and collaboration of the centers. Detailed explanations of standard operating procedure amendments and their purpose help in attracting the attention of the collaborators to the updates and facilitate their compliance with the new or changed procedures. Providing guidance about how to improve data quality and offering partnership increase the efficiency of teamwork.
Ancillary study updates	The project management team provides insight into the ancillary study proposal submission and the approval process. They extend guidance regarding approved ancillary study proposals.	Being transparent in the ancillary proposal evaluation process helps with the building of trust within the study team. Offering guidance as necessary increases efficiency and strengthens teamwork.
Pediatric update	The VIRUS: COVID-19 Registry Pediatric Team provides detailed information regarding the current status of pediatric data. They highlight pediatrics-specific data points.	Providing pediatric-specific data during the general meeting strengthens the harmony between adult and pediatric sites.
Question and answer sessions	More than half of weekly meetings is reserved for the question and answer session. The participants get an opportunity to inquire about any unclear points, and receive explanations directly from the study principal investigator. Fruitful discussions take place between the study coordinating team and participating researchers.	The chance to have a personal discussion with the primary study team allows any issues to be clarified promptly. Additionally, because all investigators are a part of the conversation, it helps them to address the same situations quickly as they encounter them. During these conversations the primary study team has the opportunity to get direct feedback from other investigators, which leads to adjustments of the project according to the needs of the researchers.

^a^VIRUS: Viral Infection and Respiratory Illness Universal Study.

##### Social Media Management

During these times of rapid information exchange, proper utilization of social media could be a great asset in such a large-scale global pandemic registry. The early recruitment of like-minded and motivated key team members could be done via professional social media platforms such as LinkedIn and Twitter. To enroll newer sites, the word could be spread through regular posts and tweets. A handful of social media–savvy champions to repost, reshare, and retweet with tagging and adding pertinent stakeholders would be a game changer. In our case, an early twitter handle (@covid19registry) was established even before the first site enrollment. On Twitter, more than 425 tweets have been sent in the first 8 months of registry initiation, accumulating over 725 followers. A robust social media presence would be a great strength for a global pandemic registry.

## Results

**Figure 4:**
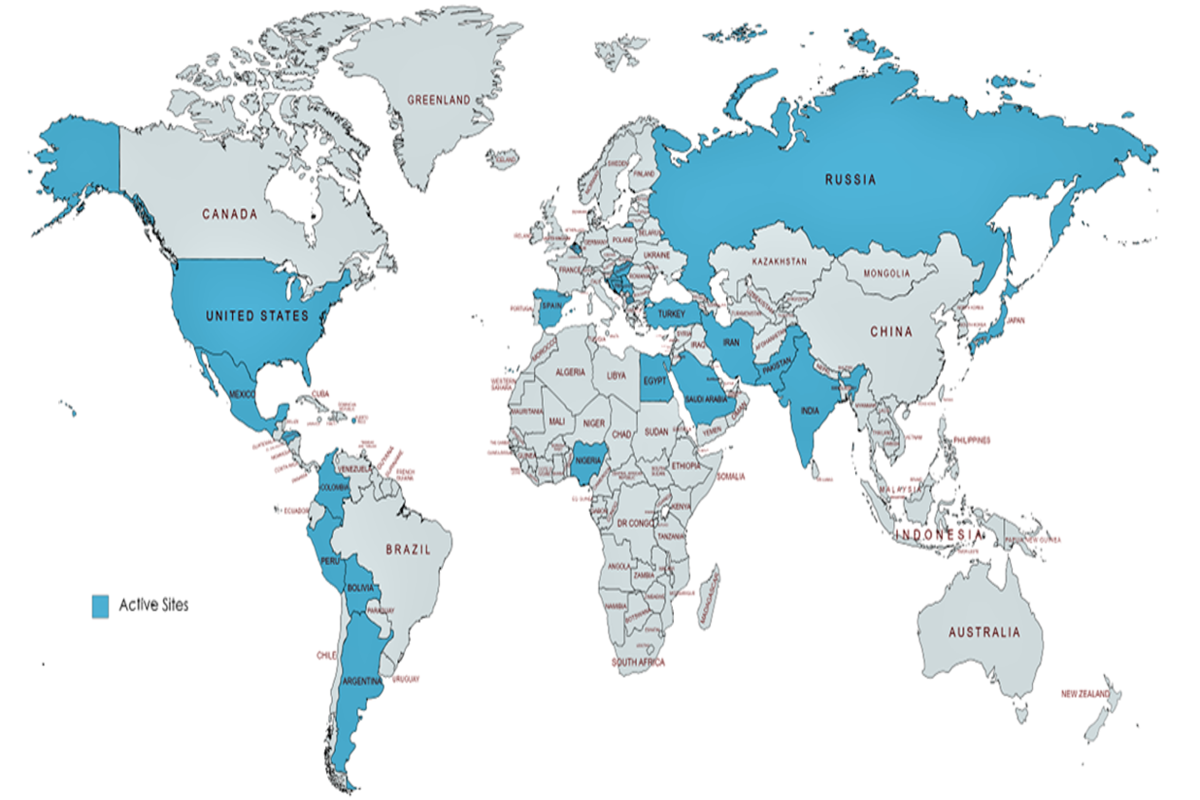
Active sites for the VIRUS: COVID-19 registry with the executive regional Leads.

The registry was established in 15 days from the inception of idea. Within the first 2 weeks, data agreements were submitted for 250 sites (Figure 4) and approval for 45 sites was achieved. A total of 69, 41, and 11 sites were enrolled in the first 3 months, respectively. Enrollment in the pediatrics registry also started from June 2020.The trend of patient enrollment is shown in Figure 2. The robust data automation management saved significant time over abstraction or manual data import processes. A total of 25 sites participate in data automation.

The extensive data that are being collected within the scope of this study would help answer some fundamental questions about this new disease. In the end it will not only be useful for determining the impact of treatment strategies, but also could provide more insight into the pathogenesis. For this purpose, researchers that are involved in the study were given the opportunity to share their research questions with the core team. After a careful review, ancillary study proposals were approved to be conducted on the collected data and they are ongoing. The large database allows for further studies on observational outcomes. In addition, we periodically gathered queries related to VIRUS Registry REDCap and suggestions to improve the registry. We also updated REDCap fields accordingly in a weekly manner after acquiring the approval of the core committee. This process will also allow reconciling large amounts of data into concise targeted information summaries and reports for statistical analysis.

## Discussion

In times of crisis, agile project management helped health care organizations form a self-organized team to set up a global registry, which implemented clear key metrics, and fostered collaboration among sites as well as sharing of resources. It also boosted quality output and improvised changes. Project management for a global registry requires rigorous training and monitoring on a large scale. We adopted the “hub and spoke” model of team organization. This style allows for more effective communication among the teams and is more adaptable to change. Establishing scope of relevant activities and assembling governance that has the right background, knowledge, and skills assured prompt response to barriers. Stakeholder analysis helped in forming alliance with shared understanding of objectives. The user interface of REDCap allowed for a quick initiation and formulation of data variables. It also proved to be an ideal tool for data import as 25 sites were using data automation. Its robust analytics helped in finding missing data and outliers. However, user maintenance is difficult in collaborating with other organizations. Robust project management was needed for successful launch of registry, which includes but not limited to, project plan, project charter, virtual meeting for project progression, and social media engagement. Our 5 key lessons learned were (1) preparation and anticipation of site needs, (2) regular communication with participating sites, (3) proactive data quality, (4) development of contingency plans through SOP, and (5) expressions of appreciation to participating sites on virtual meetings. This study can inform the project management implementation of future complex global registries.

Many studies in the past have provided guidance regarding trial management [7,18]. However, there is a lack of project management guidelines for a global registry. To our knowledge, we are the first to apply the agile project management approach to describe the conduct of a multicenter global COVID-19 registry. These results may help inform the planning of realistic clinical registry activities and set milestones for participating sites for another multicenter observational registry.

In conclusion, project management from initiation to execution with rapid availability of results in a global pandemic registry project is a herculean task. Recognizing the importance of implementing the VIRUS Registry to share best medical practices pushes the boundaries of project management and challenges traditional project management methodologies (agile in our case) during rapid implementation. However, as illustrated in this protocol, it is achievable. A well-organized database that is led by a database manager is imperative to storing patient data securely and without errors. Perhaps the most important lesson we have learned is that the success of a registry of this scope depends crucially on the willingness of health care community to contribute to a joint initiative for a common good. This project will facilitate national consensus on data standardization and subsequent automation for rapid critical care trials and national registries.

## Appendix

Multimedia Appendix 1 Global pandemic registry team organization template.

## Abbreviations

DAG: data access group
ISARIC: International Severe Acute Respiratory and emerging Infection Consortium
PEEP: Practical EHR (electronic health record) Export Pathways
REDCap: Research Electronic Data Capture
SCCM: Society of Critical Care Medicine
SOP: standard operating procedure
VIRUS: Viral Infection and Respiratory Illness Universal Study
WHO: World Health Organization
