# Supplementary figures and images for: Lessons From a Rapid Project Management Exercise in the Time of Pandemic: Methodology for a Global COVID-19 VIRUS Registry Database
Source: JMIR Res Protoc. 2022 Mar 15;11(3):e27921. doi: 10.2196/27921 (PMC8929407; doi:10.2196/27921)

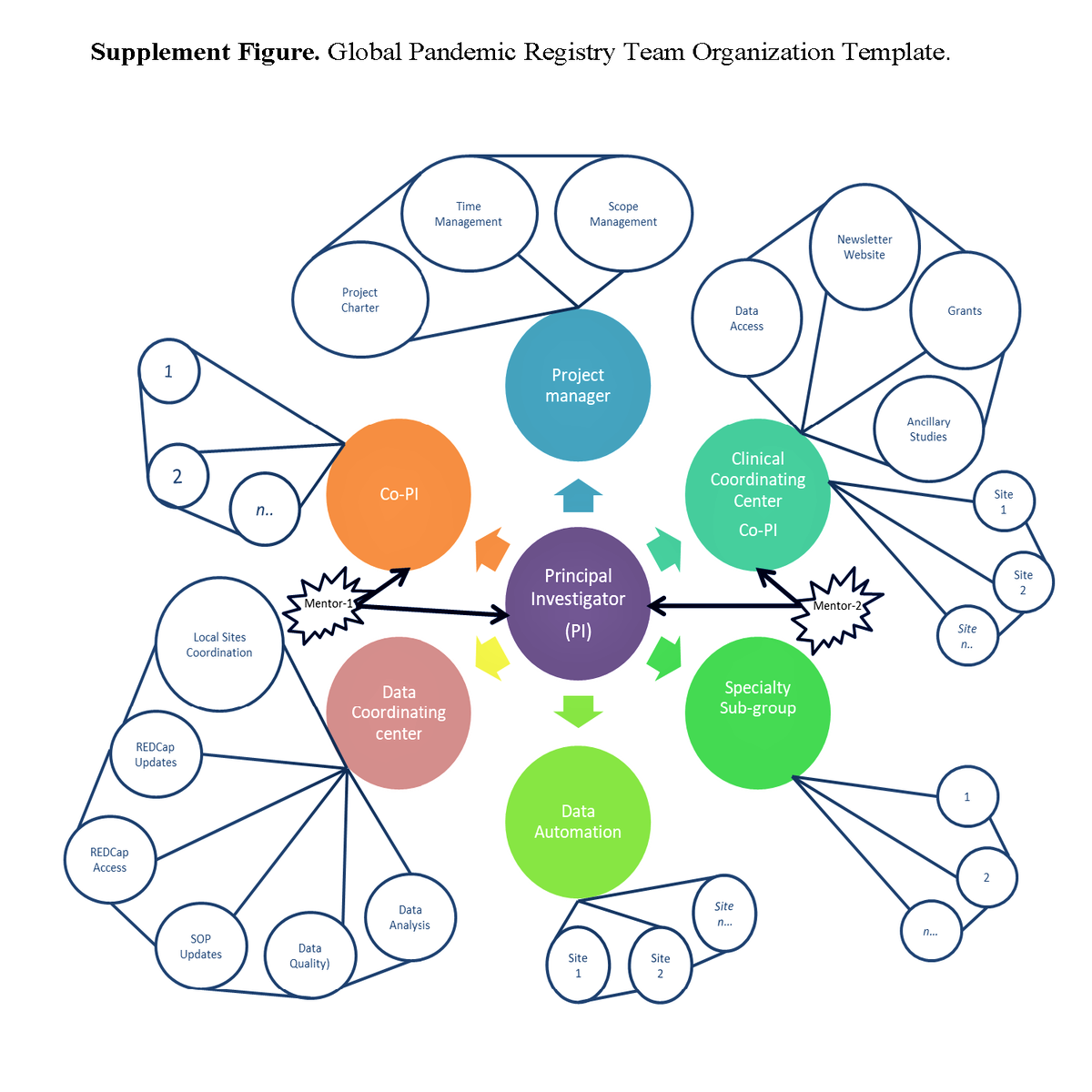

Supplement: Multimedia Appendix 1 [file resprot_v11i3e27921_app1.png]
